# Copy number gain of MET gene with low level in a metastatic lung adenocarcinoma patient represents response to salvage treatment with savolitinib and osimertinib: a case report

**DOI:** 10.3389/fonc.2025.1507677

**Published:** 2025-04-29

**Authors:** Jian Wang, Xinying Dong, Yangxin Liu, Keying Lin, Jianxin Chen

**Affiliations:** ^1^ M.D. Department of Medical Oncology, International Ward, The Quzhou Affiliated Hospital of Wenzhou Medical University, Quzhou People’s Hospital, Quzhou, Zhejiang, China; ^2^ M.D. The Third Clinical Medical College of Zhejiang Chinese Medical University, Hangzhou, Zhejiang, China; ^3^ Changsha Medical University, Changsha, Hunan, China

**Keywords:** savolitinib, osimertinib, MET amplification, efficacy, case report

## Abstract

**Background:**

Mesenchymal–epithelial transition (MET) amplification is one of the molecular mechanisms of abnormal MET oncogenic signaling in non-small cell lung cancer (NSCLC), significantly contributing to tumor cell survival, proliferation, metastasis, and drug resistance. The results of the TATTON trial showed that the combination of savolitinib and osimertinib can prolong the survival of patients with advanced EGFR-TKI-resistant NSCLC and high-level acquired MET amplification.

**Case presentation:**

We present a case of an NSCLC patient who exhibited acquired MET amplification with a gene copy number (GCN) of 3 following resistance to EGFR-TKI. The patient achieved a substantial response to salvage therapy with savolitinib and osimertinib, resulting in a 7-month progression-free survival (PFS).

**Conclusions:**

We considered that a regimen of savolitinib + osimertinib combination sometimes may still be potentially beneficial for NSCLC patients with low-GCN-level MET amplification. However, it needs further confirmation in a larger cohort.

## 1 Introduction

Mesenchymal–epithelial transition (MET) gene, located on chromosome 7q31, is an oncogenic receptor tyrosine kinase for hepatocyte growth factor (HGF). The activation of the MET pathway by MET amplification plays important roles in tumor cell survival, proliferation, metastasis, and drug resistance (1). There are approximately 10% to 15% of patients with non-small cell lung cancer (NSCLC) harboring MET amplification as a secondary driver of acquired resistance to targeted therapy including EGFR-TKIs or Anaplastic Lymphoma Kinase-Tyrosine Kinase Inhibitors (ALK-TKIs) (2, 3). Beyond genetic alterations, the tumor microenvironment (TME) plays a crucial role in MET amplification-driven oncogenesis and treatment resistance. Cancer-associated fibroblasts (CAFs) are a major source of HGF, which can sustain MET signaling in a paracrine manner, leading to persistent oncogenic activation even in the presence of EGFR-TKIs (4, 5). Studies have shown that stromal HGF secretion from CAFs enables MET-amplified tumor cells to bypass MET inhibition, thereby reducing the efficacy of MET-targeted therapies. Additionally, interactions between MET and integrin-mediated adhesion to the extracellular matrix (ECM) further contribute to tumor invasiveness and resistance to therapy (6). To address that, recent clinical researchers have shown that NSCLC patients with acquired MET amplification may benefit from MET-TKIs including crizotinib (7), capmatinib (8), savolitinib (9), and tepotinib (10). In the TATTON study, the landmark study for savolitinib in MET-amplification NSCLC, only patients with MET-amplification gene copy number (GCN) of more than 5 were enrolled (11). As a result, objective response rate (ORR) trended higher in tumors with MET GCN ≥ 10 (34%; 95% CI, 18–54) versus tumors with MET GCN 5 to 9 (25%; 95% CI, 10–47), which suggested that savolitinib + osimertinib combination is more effective in higher MET GCN levels (11). However, there have been few works of literature investigating the efficacy of MET-TKIs in NSCLC patients with acquired MET amplification with a low GCN level (GCN < 5) in capmatinib, savolitinib, or tepotinib. The clinical demand of NSCLC patients with low-GCN-level MET amplification has not been met. Herein, we present a case of NSCLC patient harboring acquired MET amplification with a low GCN level (GCN = 3) after EGFR-TKI resistance achieving substantial response to savolitinib + osimertinib combination, hence resulting in progression-free survival (PFS) time reached of 7 months. We considered that a regimen of savolitinib + osimertinib combination sometimes may still be potentially beneficial for NSCLC patients with low-GCN-level MET amplification, which may provide some thought-provoking ideas in clinical practice.

## 2 Case presentation

**Figure 1:**
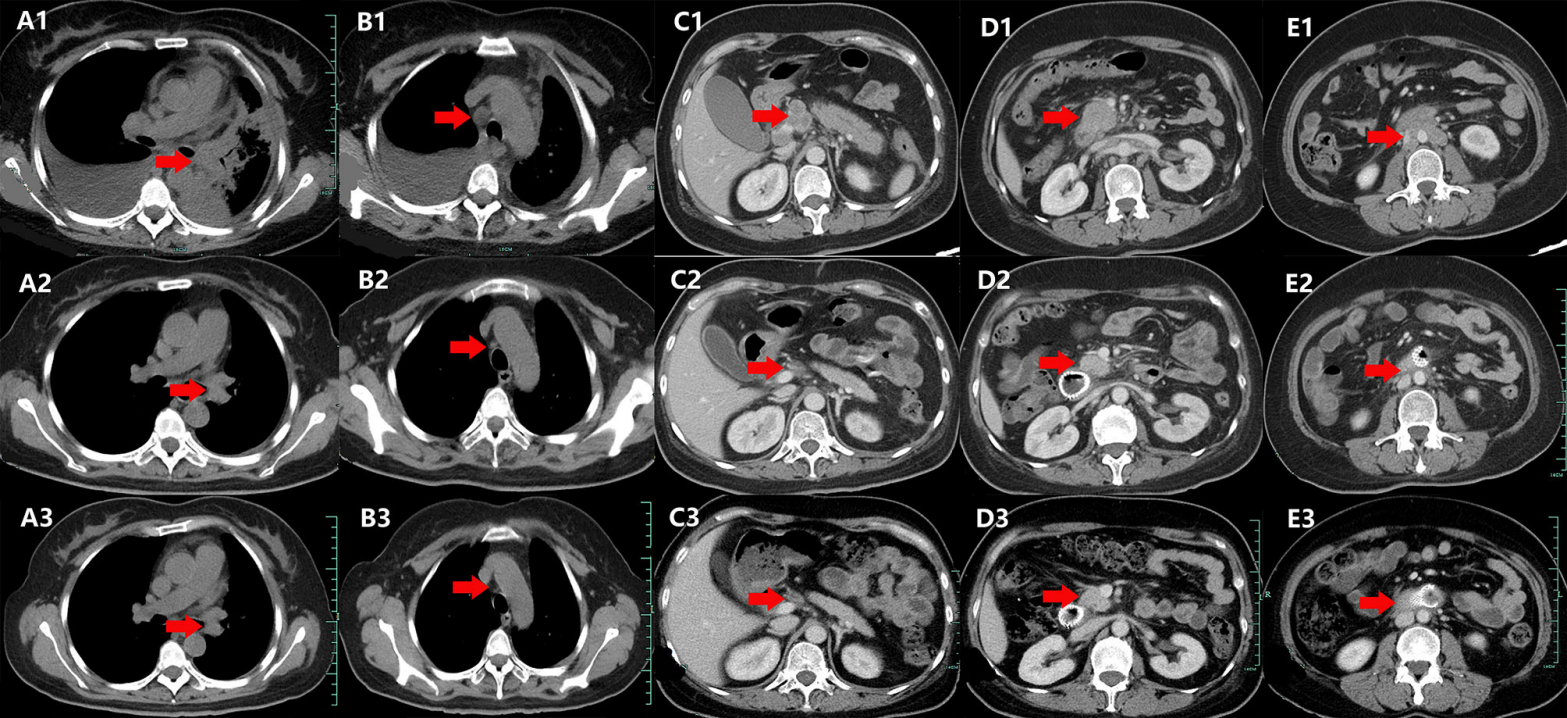
Variations of a space-occupying lesion on retroperitoneum, mediastinum, and left hilar area by CT images during the treatment (red arrowheads). **(A1–E1)** February 2022. **(A2–E2)** April 2020. **(A3–E3)** July 2022.

**Figure 2:**
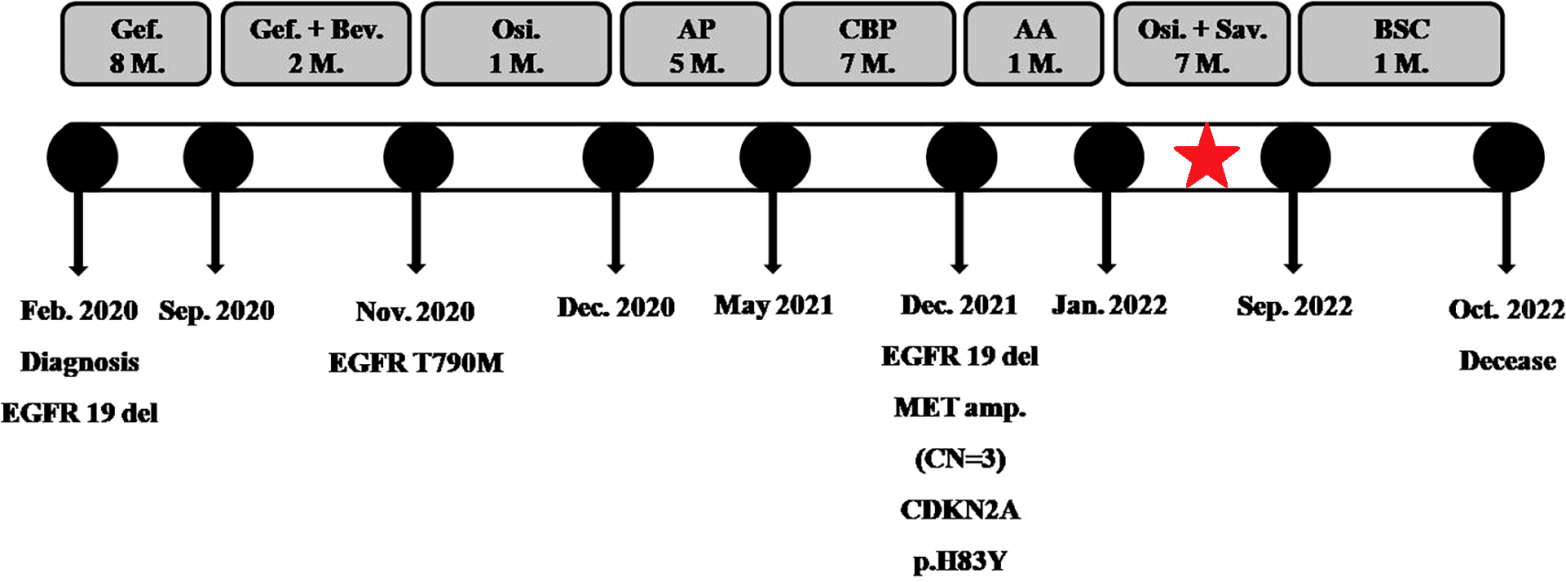
The overview of the approaches to diagnosis, treatment regimens, and PFS for each regimen. The red pentagram denotes patients who received salvage therapy with a combination of MET-TKI and EGFR-TKI. M, month; Gef, gefitinib; Bev, bevacizumab; Osi, osimertinib; AP, cisplatin + pemetrexed; CBP, carboplatin + bevacizumab + albumin-bound paclitaxel; AA, abemaciclib + anlotinib; Sav, savolitinib; BSC, best supportive care; PFS, progression-free survival.

A 44-year-old Chinese woman was admitted to the hospital on January 10, 2020, with lumbodorsal pain for 2 months. The patient denied smoking or alcohol intake history. In addition, she denied any other medical or family history either. An abdominal computed tomography (CT) scan on January 17, 2020, showed a space-occupying lesion on the right lumbar, along with multiple lesions on bilateral adrenalectomy suggesting a metastatic tumor. Simultaneously, a chest CT scan revealed a space-occupying lesion on the left inferior lobe, as well as lymph node enlargement in the mediastinum and left hilar area, with involvement of the lower left pulmonary artery. Furthermore, there was no other distant metastatic lesion detected according to brain MRI findings. Subsequently, she received a puncture biopsy for right lumbar lesion, the results of which revealed metastatic adenocarcinoma most likely originating from the lung according to the imaging findings, and immunohistochemistry outcomes presented as follows: TTF-1 (positive), Napsin A (positive), P40 (negative), P63 (negative), CK5/6 (negative), CK (AE1/AE3) (positive), Syn (negative), CgA (negative), Ki-67 15% (positive), and programmed cell death ligand-1 (PD-L1) negative. Furthermore, next-generation sequencing (NGS) was performed using tissue sample (tumor cellularity 70%, including 168 cancer-related genes; Burning Rock, Beijing, China) showed EGFR exon 19 p.E746_S752delinsV in-frame deletion (frequency as 30.67%) caused by c.2237_2255delinsT mutation. Based on those, the patient was diagnosed with left lung adenocarcinoma, with involvement of the lower left pulmonary artery, along with metastasis on lymph node in the mediastinum and left hilar area, requiring right lumbar and bilateral adrenalectomy, which was staged as IVB (cT4N2M1) according to the criteria of the American Joint Committee on Cancer (AJCC) 8th edition. Her initial treatment regimen was started with monotherapy with gefitinib, an oral first-generation EGFR-TKI. The best response for the target therapy was presented as partial response (PR), which lasted for 8 months. The chest CT in September 2020 showed slow progression of the primary tumor in the lung. To address that, the addition of bevacizumab, a vascular endothelial growth factor inhibitor, was prescribed in combination with gefitinib. However, rapid progression of the primary tumor in the lung was observed after merely 2 months of treatment. Subsequent osimertinib was administrated because of the detection of EGFR exon 20 T790M mutation by NGS with plasma sample. Nonetheless, the salvage treatment with osimertinib finally resulted in the rapid progression of the primary tumor and metastatic lymph nodes only 1 month later in December 2020. Chemotherapy with an AP regimen (cisplatin 75 mg/m^2^ on day 1 and pemetrexed 500 mg/m^2^ on day 1, every 21 days) was adopted as a systematic choice. The progression disease (PD) status was detected on May 2021, followed by another cytotoxic regimen with CBP (carboplatin AUC 5 on day 1, bevacizumab 7.5 mg/kg on day 1, and albumin-bound paclitaxel 260 mg/m^2^ on day 1, every 21 days). An adverse event of neutropenia at grade 3 during the chemotherapy was detected and managed. Her cytotoxic chemotherapy was terminated in December 2021 because of the emerging enlargement of para-aortic lymph nodes and supraclavicular lymph nodes. Repeated puncture biopsy for supraclavicular lymph nodes was performed, with subsequent NGS conducted. As a result, CDKN2A, exon 2 p.His83Tyr, due to c.247C>T (frequency as 41.03%), and MET amplification (CN: 3.0) were detected. In terms of the higher frequency of a CDKN2A mutation, salvage treatment of abemaciclib, an oral CDK4/6 inhibitor, combined with anlotinib (an oral antiangiogenic TKI), was suggested as a palliative choice, which was also agreed by the patient herself. However, rapid progression of the primary tumor and metastatic lesions was observed again. Her performance status (PS) deteriorated to 4, with obstructive jaundice emerging after 1-month treatment. As palliative therapy, percutaneous transhepatic cholangial drainage was performed to relieve jaundice. The patient felt bad, with severe cachexia. However, because she was so young, her family did not want to give up salvage treatment. She thereby received savolitinib (an oral MET TKI) 400 mg once per day, combined with osimertinib 80 mg once per day as a further-line attempt. Wondrously, her PS score improved from 4 to 2 after only a 2-week exposure. Repeated chest and abdominal CT scans in February (**Figures 1A1–E1**), April (**Figures 1A2–E2**), and July (**Figures 1A2–E2**) 2022 revealed partial response of the primary and metastatic lesions, and jaundice was relieved significantly. The efficacy of that salvage treatment lasted for 7 months and finally became resistant in September 2022. There was no treatment-related adverse event observed during the exposure to that regimen. She died of a progression of lung cancer in October 2022, with an overall survival time of 32 months. The overview of the approaches to diagnosis, treatment regimens, and PFS for each regimen is presented in **Figure 2**.

## 3 Discussion

In this report, we present a case of an NSCLC patient who developed acquired MET amplification with a GCN of 3 following resistance to EGFR-TKI treatment. The patient exhibited a substantial response to salvage therapy with savolitinib and osimertinib, achieving a PFS of 7 months.

MET amplification is a key molecular mechanism underlying aberrant MET oncogenic signaling in NSCLC, significantly contributing to tumor cell survival, proliferation, and metastasis. Moreover, acquired MET amplification is a well-recognized resistance mechanism to EGFR-TKI therapy, with a reported prevalence of 5%–21% following first- or second-generation EGFR-TKI treatment, 7%–15% after first-line osimertinib therapy, and 5%–50% after second-line or subsequent osimertinib treatment (12). Given these findings, numerous studies have explored the efficacy of the combination of EGFR-TKIs with MET-TKIs in advanced NSCLC patients harboring secondary MET amplification, achieving satisfactory therapeutic outcomes. Liu et al. conducted a large real-world study to evaluate the efficacy of three treatment strategies in 70 patients with acquired MET amplification, as identified by NGS following EGFR-TKI therapy. The treatment strategies included EGFR-TKI plus crizotinib (n = 38), crizotinib monotherapy (n = 10), and chemotherapy (n = 22) (13). A GCN cutoff of 2.25 was applied. The results demonstrated that patients receiving the dual TKI regimen had a significantly longer PFS compared to those treated with crizotinib monotherapy or chemotherapy (5.0 vs. 2.3 vs. 2.9 months, p = 0.010). Furthermore, the INSIGHT study, an open-label, multicenter, randomized trial (14), evaluated 19 patients with advanced NSCLC who had previously failed EGFR-TKI therapy and exhibited MET amplification. These patients were randomized to receive either tepotinib in combination with gefitinib or standard platinum-based doublet chemotherapy. The diagnosis of MET amplification was confirmed using fluorescence *in situ* hybridization (FISH), with criteria set at a GCN of ≥5 or a MET/CEP17 ratio of ≥2. The results indicated that the combination of tepotinib and gefitinib had a longer PFS (HR 0.13, 90% CI 0.04–0.43) and OS (HR 0.10, 90% CI 0.02–0.36) compared to chemotherapy. The ORR was 66.7% in the combination TKI group, compared to 42.9% in the chemotherapy group. Similarly, the TATTON trial evaluated 138 patients with EGFR-mutant, MET-amplified, or MET-overexpressing NSCLC who were treated with a combination of savolitinib and osimertinib (11). The MET amplification was defined as MET GCN ≥ 5 by NGS or MET/CEP7 ratio ≥ 2 by FISH. The patients were categorized into three cohorts based on their history of osimertinib treatment and T790M expression. Across all cohorts, partial responses were observed, with ORRs ranging from 33% to 65% and a median PFS of 5.5 to 11.1 months. Additionally, the ongoing ORCHARD study has enrolled 20 patients who developed acquired MET amplification following osimertinib monotherapy (15). These patients received second-line treatment with a combination of osimertinib and savolitinib, resulting in a partial response rate of 41%.

Osimertinib is currently recommended as the first-line treatment for advanced EGFR-positive NSCLC. However, MET amplification, as previously described, is not uncommon in patients treated with osimertinib and is recognized as the most prevalent mechanism of resistance to osimertinib identified. As illustrated in **Figure 2**, the patient also developed MET amplification (GCN = 3) following multiple lines of treatment, including osimertinib. Given the patient’s strong desire to survive and the findings from the HATTON trial, she received savolitinib at a dosage of 400 mg once daily and osimertinib at 80 mg once daily as a subsequent treatment attempt. This combination resulted in a PFS time of 7 months. In a previously published *in vitro* study, the researchers established EGFR-mutant NSCLC cell lines that had acquired resistance to EGFR-TKIs and discovered the presence of MET amplification in these resistant cell lines (16). Notably, the combined inhibition of MET and EGFR signaling using MET-TKIs and EGFR-TKIs successfully restored drug sensitivity in these models. The Phase I trial, PROFILE 1001, evaluated crizotinib in 38 patients with advanced NSCLC harboring MET amplification, as diagnosed by FISH. The patients were categorized based on their MET/CEP7 ratios into the high (MET/CEP7 ≥ 4; n = 21), medium (2.2 < MET/CEP7 < 4; n = 14), or low (1.8 ≤ MET/CEP7 ≤ 2.2; n = 3) amplification groups. The results indicated that one of the three patients with low-level MET amplification exhibited a partial response, with a median PFS of 1.8 months. Therefore, the authors proposed that the therapy of savolitinib and osimertinib can prolong the survival of patients with advanced EGFR-TKI-resistant NSCLC and low-level acquired MET amplification. Furthermore, integrating single-cell data analysis and machine learning to build prognostic models may provide valuable insights into predicting the clinical outcomes of NSCLC patients with low-level MET amplification undergoing targeted therapy, thereby optimizing personalized treatment strategies (17, 18).

There are some limitations in the present case. NGS provides a comprehensive analysis of MET amplification and other treatment-related genetic alterations, making it widely employed in the precision management of NSCLC. However, there is currently no standardized cutoff value or grading criteria for MET amplification diagnosed based on NGS, leading to variability in cutoff values across different studies. For instance, the cutoff value for MET amplification was defined as GCN ≥ 5 in the TATTON study (11), GCN ≥ 2.3 in the INC280 study (8), and GCN ≥ 6 in the INSIGHT 2 study (19). Considering that the current clinical cutoff value range spans GCN 2.3 to 10, we concluded that this case exhibits low-level MET amplification based on NGS. Nonetheless, the poor concordance between low-level MET amplification identified by NGS and the results from FISH, the gold standard method for detecting MET amplification, reduced the reliability and universality of the conclusions of this study. In addition, the optimal MET-TKI for patients with advanced NSCLC with low-level acquired MET amplification also needs further study.

Briefly, we present a case of an NSCLC patient with acquired MET amplification at a low GCN level (GCN = 3) following resistance to EGFR-TKI, achieving a notable response to the combination of savolitinib and osimertinib. This treatment resulted in a PFS of 7 months. We considered that a regimen of savolitinib + osimertinib combination sometimes may still be potentially beneficial for NSCLC patients with low-GCN-level MET amplification, which may provide some thought-provoking ideas for the treatment options for NSCLC patients with acquired low-level MET amplification and effectively make up for the shortcomings of the HATTON test in patients with low-level MET amplification.

## Data Availability

The original contributions presented in the study are included in the article/supplementary material. Further inquiries can be directed to the corresponding author.
